# Structural and Genomic Evolution of RRNPPA Systems and Their Pheromone Signaling

**DOI:** 10.1128/mbio.02514-22

**Published:** 2022-10-19

**Authors:** Alonso Felipe-Ruiz, Alberto Marina, Eduardo P. C. Rocha

**Affiliations:** a Instituto de Biomedicina de Valencia (IBV), CSIC and CIBER de Enfermedades Raras (CIBERER), Valencia, Spain; b Institut Pasteur, Université de Paris Cité, CNRS UMR3525, Microbial Evolutionary Genomics, Paris, France; Duke University School of Medicine

**Keywords:** RRNPPA family, TPR domain, peptide pheromone, quorum sensing, signal transduction, transcription factors

## Abstract

In *Firmicutes*, important processes such as competence development, sporulation, virulence, and biofilm formation are regulated by cytoplasmic quorum sensing (QS) receptors of the RRNPPA family using peptide-based communication. Although these systems regulate important processes in a variety of bacteria, their origin and diversification are poorly understood. Here, we integrate structural, genomic, and phylogenetic evidence to shed light on RRNPPA protein origin and diversification. The family is constituted by seven different subfamilies with different domain architectures and functions. Among these, three were found in *Lactobacillales* (Rgg, ComR, and PrgX) and four in *Bacillales* (AimR, NprR, PlcR, and Rap). The patterns of presence and the phylogeny of these proteins show that subfamilies diversified a long time ago, resulting in key structural and functional differences. The concordance between the distribution of subfamilies and the bacterial phylogeny was somewhat unexpected, since many of the subfamilies are very abundant in mobile genetic elements, such as phages, plasmids, and phage-plasmids. The existence of diverse propeptide architectures raises intriguing questions about their export and maturation. It also suggests the existence of diverse roles for the RRNPPA systems. Some systems encode multiple pheromones on the same propeptide or multiple similar propeptides, suggesting that they act as “chatterers.” Many others lack pheromone genes and may be “eavesdroppers.” Interestingly, AimR systems without associated propeptide genes were particularly abundant in chromosomal regions not classed as prophages, suggesting that either the bacterium or other mobile elements are eavesdropping on phage activity.

## INTRODUCTION

Bacteria use diffusible chemical messengers, termed pheromones, to mediate intercellular communication in a process called quorum sensing (QS) (1). Signaling by QS systems occurs in distinct steps: pheromone production, maturation and externalization, signal sensing by membrane attached receptors, and reinternalization and modulation of cytoplasmatic receptors. Proteins of the RRNPPA family are the most frequent pheromone receptors in *Firmicutes* and use small secreted oligopeptides as pheromones (2). The RRNPPA family received its name from the founding members: Rap phosphatases from *Bacillus* species, Rgg from Streptococcus species, NprR from the Bacillus cereus group, PlcR from the Bacillus cereus group, PrgX from Enterococcus faecalis, and AimR from *Bacillus* phages (3, 4). It may also include families whose representatives are ComR from the Streptococcus group (5, 6), AloR from the *Paenibacillus* group (7), QsrR from Clostridium acetobutylicum (8), and TrpA from the Streptococcus group (9). AimR, Rap, and NprR proteins were predominantly found in *Bacillales*, whereas Rgg and PrgX homologs were described for *Lactobacillales*. Previous studies identified PlcR in both *Bacillales* and *Lactobacillales* (1). Hence, the complexity of QS, combined with the diversity of cell structures and environments, led to the emergence of different but related systems (10).

**FIG 1:**
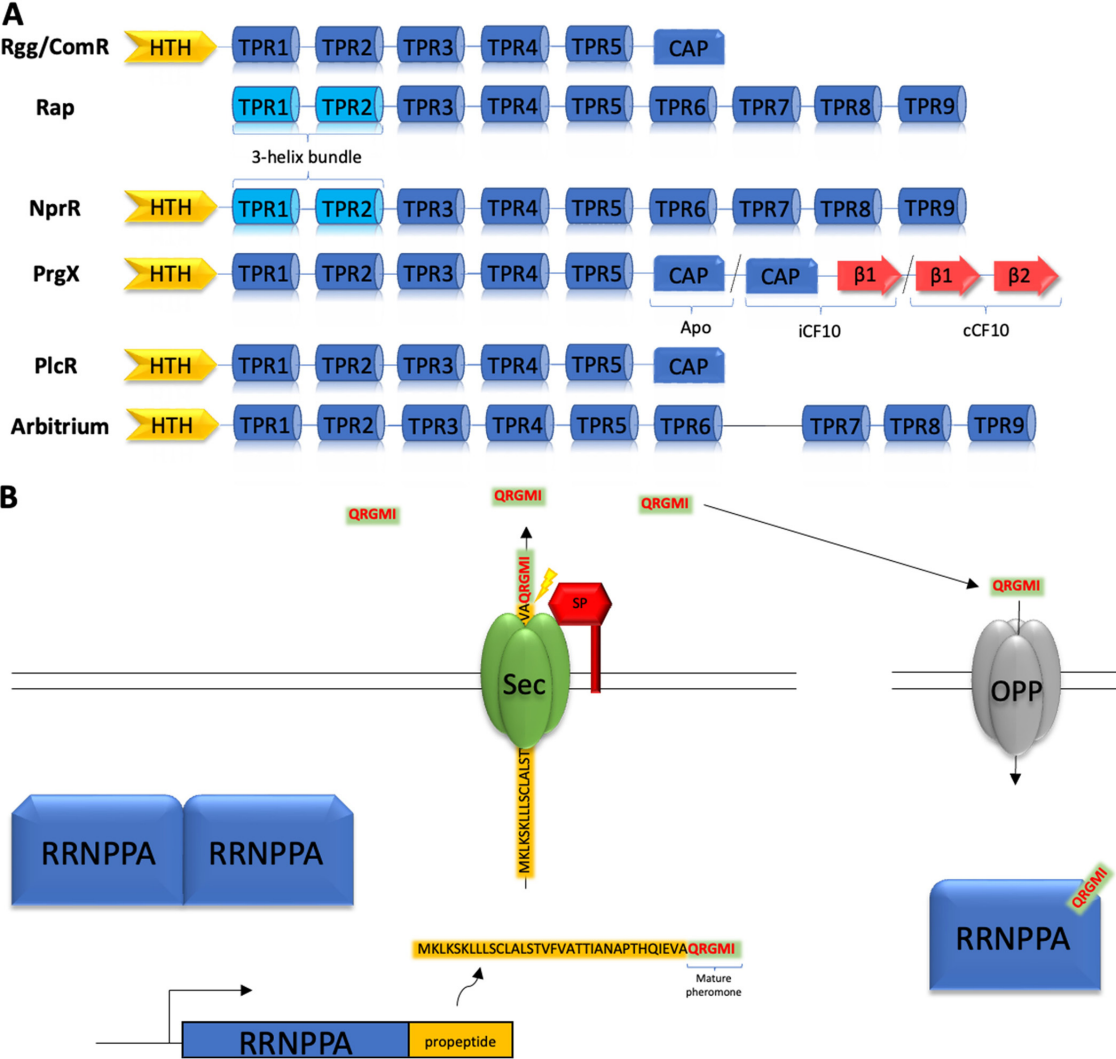
Architecture of the members of RNNPPA family and signaling process. (A) Domain architecture of the different subfamilies of RRNPPA proteins. (B) Diagram of the signaling process in the RRNPPA family. The QS propeptide is encoded immediately upstream or downstream of the RRNPPA gene. When the propetide encodes a single peptide pheromone, the mature QS pheromone (located at the C-terminal region of the propeptide) is created by an N-terminal cut performed by the signal peptidase (SP) and exported by the SEC translocon. Mature QS pheromone is internalized by the oligopeptide permease (OPP); cytoplasmic pheromone can bind to the RRNPPA receptor, modulating its function.

The RRNPPA family is characterized by the presence of 5 to 9 tandem tetratricopeptide repeat (TPR) motifs, which are ~34 amino acids (aa) long and fold as a pair of antiparallel helices (Fig. 1). They form a right-handed superhelical structure with a concave internal surface where the binding pocket for the regulatory peptide is located. Rap, NprR, PlcR, and AimR have canonical or degenerated TPR sequences. In contrast, Rgg and PrgX proteins do not contain recognizable TPR sequences but adopt TPR-like folding. The N-terminal region of the RRNPPA protein is the most variable. Rgg, NprR, PlcR, PrgX, and AimR act as transcriptional regulators and carry an N-terminal helix-turn-helix (HTH) DNA binding domain at this position, while Rap and NprR have a three-helix bundle (3HB) implicated in protein-protein interaction (Fig. 1A). The presence of both the 3HB and HTH DNA binding domain in NprR has led to suggestions that it is an evolutionary intermediate between Rap proteins and the other members of RRNPPA family (11).

Most RRNPPA families are transcriptional regulators. NprR and PlcR from the B. cereus group control the expression of genes required for necrotrophic and virulence lifestyles of the bacteria (12, 13), respectively. NprR also regulates sporulation by dephosphorylation of Spo0F (13–17). Rgg regulators are prevalent in several streptococci. Streptococcus pyogenes encodes four Rgg paralogs regulating different bacterial processes, such as biofilm formation and virulence (18, 19). ComR, a Rgg-like regulator, controls a cryptic competence pathway in S. pyogenes (5). PrgX from Enterococcus faecalis, regulates conjugation of the antibiotic resistance plasmid pCF10 (20). Finally, AimR from *Bacillus* phages regulates lysis-lysogeny decisions in temperate phages infecting *Bacillus* species. They allow the phage to make informed choices of when to enter and when to leave lysogeny (21–23). Several mobile genetic elements (MGEs), including conjugative elements and phages, employ short-range communication to assess the fraction of susceptible host cells in their vicinity and adaptively trigger horizontal gene transfer in response (24). Rap proteins from *Bacillus* species, in contrast with the other members of the RRNPPA family, are not transcriptional regulators. They regulate two-component systems by blocking competence regulator ComA DNA interaction or by dephosphorylation of the sporulation factor Spo0F (25, 26). Beyond their role in competence and sporulation, some Rap proteins regulate functions of MGEs. For example, a Rap present in plasmid pLs20 regulates plasmid conjugation (27), and RapI regulates excision and transfer in the ICEBs1 element (28, 29). Rap proteins have also been found in prophages (30).

The pheromones are translated as propeptides, secreted outside of the cell, and then processed to generate a mature small peptide that can be detected by membrane-attached histidine kinases or reinternalized and bound by RRNPPA receptors (31). The binding of the QS pheromone induces a conformational change affecting both TPR and N-terminal domains that modifies the affinity between the RRNPPA receptors and their cognate partners (32). This conformational change can induce protein inhibition or activation, depending on the system. Moreover, systems of the PrgX family encode two different peptides, one activator of PrgX activity and one with an inhibitory effect (33).

The mechanisms of communication between RRNPPA QS systems are diverse and complex. The gene for the pheromone is usually immediately upstream or downstream of the gene encoding the RRNPPA protein. However, there are many variants to the archetypical genetic structure of one RRNPPA receptor preceded/followed by one pheromone gene. The pheromones of PrgX are usually not encoded in the vicinity of the gene (34). Those of Rap proteins are sometimes present in more than one copy. These duplicates could facilitate the diversification of the peptide-receptor specificity: they allow keeping of one copy of the original peptide while the other can diverge and gain novel functions (35). The opposite situation was also described for Rap systems, i.e., QS regulators lacking a peptide pheromone (36, 37). For example, the RapB system from Bacillus subtilis 168 lacks an open reading frame (ORF) coding for a propeptide. Instead, it recognizes the PhrC pheromone associated with RapC, thereby allowing control of sporulation and competence with one single pheromone (38). Bacillus subtilis 168 encodes two other endogenous Rap systems (RapJ and RapD) that also lack an associated pheromone. These systems can be classified as eavesdroppers, i.e., systems that respond to a signal from Rap systems without producing one. RapB, RapD, and RapJ are conserved across *Bacillus* species, suggesting that eavesdropper systems could be common.

Despite the extensive structural and biochemical work previously done on the RRNPPA family, its distribution, origin, and diversification are not well understood. The low sequence similarity and diverse repertoires of protein domains characteristic of this family have complicated these studies. In this study, we have built protein profiles to identify and discriminate these proteins and have studied their evolutionary history and clarified their distribution. We have also searched for the pheromone gene and tried to understand its features in the light of the cell secretory pathways and the ecological role of these systems in bacteria and their MGEs.

## RESULTS

### Identification and distribution of RRNPPA proteins.

To understand the prevalence of RRNPPA systems among *Firmicutes*, we collected the reference validated proteins of each RRNPPA subfamily (Rap, Rgg, ComR, NprR, PlcR, PrgX, and AimR) and used them to build seven hidden Markov model (HMM) protein profiles. We used HMM profiles to identify proteins with low sequence similarity, because previously described members of certain subfamilies show sequence similarity below 20%. When the number of described sequences was too small to build reliable HMM profiles, we supplemented the set with homologous proteins identified in the genomes (see Materials and Methods). We only added proteins encoded next to a gene matching the characteristics of a pheromone to minimize the risk of adding false positives in the sets used to build the HMM profiles. The profiles were able to identify all the known members of the subfamilies and discriminate them from the known members of the other subfamilies. The only exception concerns the Rap HMM profile, which detects some NprR proteins because they share the same core architecture (3HB and 9 TPR repetitions). The inverse does not happen, because the NprR HMM profile also includes the specific HTH DNA binding domain. Consequently, the Rap hits that also matched the NprR HMM profile were classed as NprR. The families Rgg and ComR have similar domain architectures but are only matched by their respective HMM profiles. This could be confirmed, because the nearby propeptide genes were the ones expected for each of these subfamilies (see below). A final problem concerns the Rgg model, which detected proteins from the MutR and the GadR family of transcriptional regulators. We excluded the proteins whose gene lacked a nearby gene encoding the pheromone, to avoid including the proteins not related to QS.

We searched the RefSeq database of 13,513 complete bacterial genomes, including 11,806 plasmids and 10,247 predicted prophages, for genes encoding proteins matching each of the HMM profiles. We found 3985 Rap, 658 Rgg, 282 NprR, 141 PlcR, 579 ComR, 18 PrgX, and 756 AimR homologs (Table 1). Hence, Rap proteins are the most abundant members of the RRNPPA family, followed by AimR, Rgg, and ComR, while PrgX is the least frequent subfamily. Naturally, these relative frequencies are subject to caution since the genome databases are biased toward cultivated and human-associated bacteria.

**TABLE 1:** Frequency of genomes (and RRNPPA genes) from key genera

Genus	% of *Bacilli* genomes in database	% of *Bacilli* genomes encoding RRNPPA proteins
*Bacillus*	20	78 (AimR, NprR, PlcR, Rap)
Streptococcus	20	19 (ComR, Rgg)
*Virgibacillus*	0.3	0.5 (AimR, NprR)
*Enterococcus*	5	<1 (PrgX)
Staphylococcus	19	<0.05 (Rgg)

**FIG 2:**
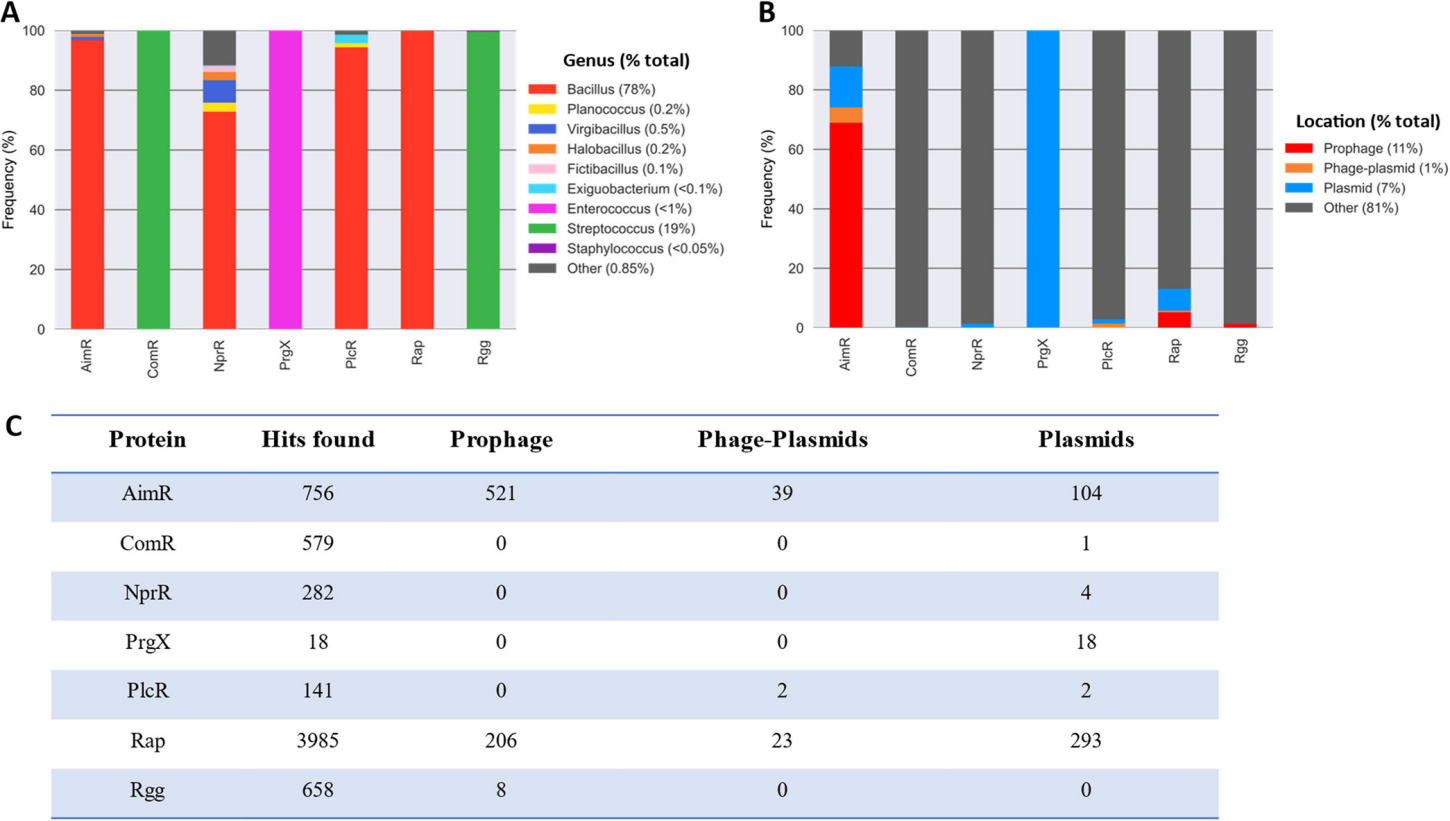
Distribution of RRNPPA proteins. (A) Distribution of RRNPPA proteins among the genera. (B) RRNPPA family distribution in MGE. Data were normalized to overcome the overrepresentation of certain clades. (C) Number of hits found in prophages, plasmids, and phage-plasmids.

The RRNPPA genes were found in several genera of the *Firmicutes*, all but one within the class *Bacilli*, with a preponderance of bacteria from the genus *Bacillus* (78% of the hits), followed by those of Streptococcus (19%). This overrepresentation in *Bacillus* cannot be explained only by the large number of available genomes for this genus, which is only 20% of the *Bacilli* genomes. In contrast, Staphylococcus spp. represent 19% of the genomes of *Bacilli* but less than 0.05% of those encoding RRNPPA proteins. Hence, *Bacilli* contain many RRNPPA proteins, but these tend to be concentrated in a few clades, most notably in *Bacillus* (Table 1). The distribution of the different families of RRNPPA proteins in the members of *Firmicutes* is far from random. Most families are present in a few clades. Rap, NprR, PlcR, and AimR proteins were found predominantly in *Bacillus* species, whereas Rgg, PrgX, and ComR were only found in *Lactobacillales*, mainly in Streptococcus spp. (Rgg and ComR) and *Enterococcus* spp. (PrgX) (Fig. 2A). The NprR subfamily is the most broadly represented, being found in 21 different genera.

The observation that the distribution of the RRNPPA subfamilies tends to follow the bacterial taxonomy might be regarded as surprising in light of the numerous reports that these proteins are often encoded by MGEs (20, 21, 27, 28). We thus searched for the proteins encoded in the MGEs present in the genomes. Notably, we searched for plasmids, prophages integrated in the chromosome, and phage-plasmids. These MGEs include 13% of the Rap hits and 87% of the AimR hits. PrgX was the only subfamily that was present only in plasmids. This suggests that PrgX acts only in plasmid conjugation (39). Since integrative conjugative elements (ICEs) are more abundant than conjugative plasmids in *Firmicutes* (40), these results also suggest that PrgX does not usually regulate conjugation of ICEs. Finally, NprR, PlcR, and ComR were occasionally found in plasmids and phage-plasmids but not in prophages (Fig. 2), while some prophages occasionally carry Rgg systems. In conclusion, MGEs often encode RRNPPA proteins but only of a few specific types.

### Evolution of RRNPPA.

**FIG 3:**
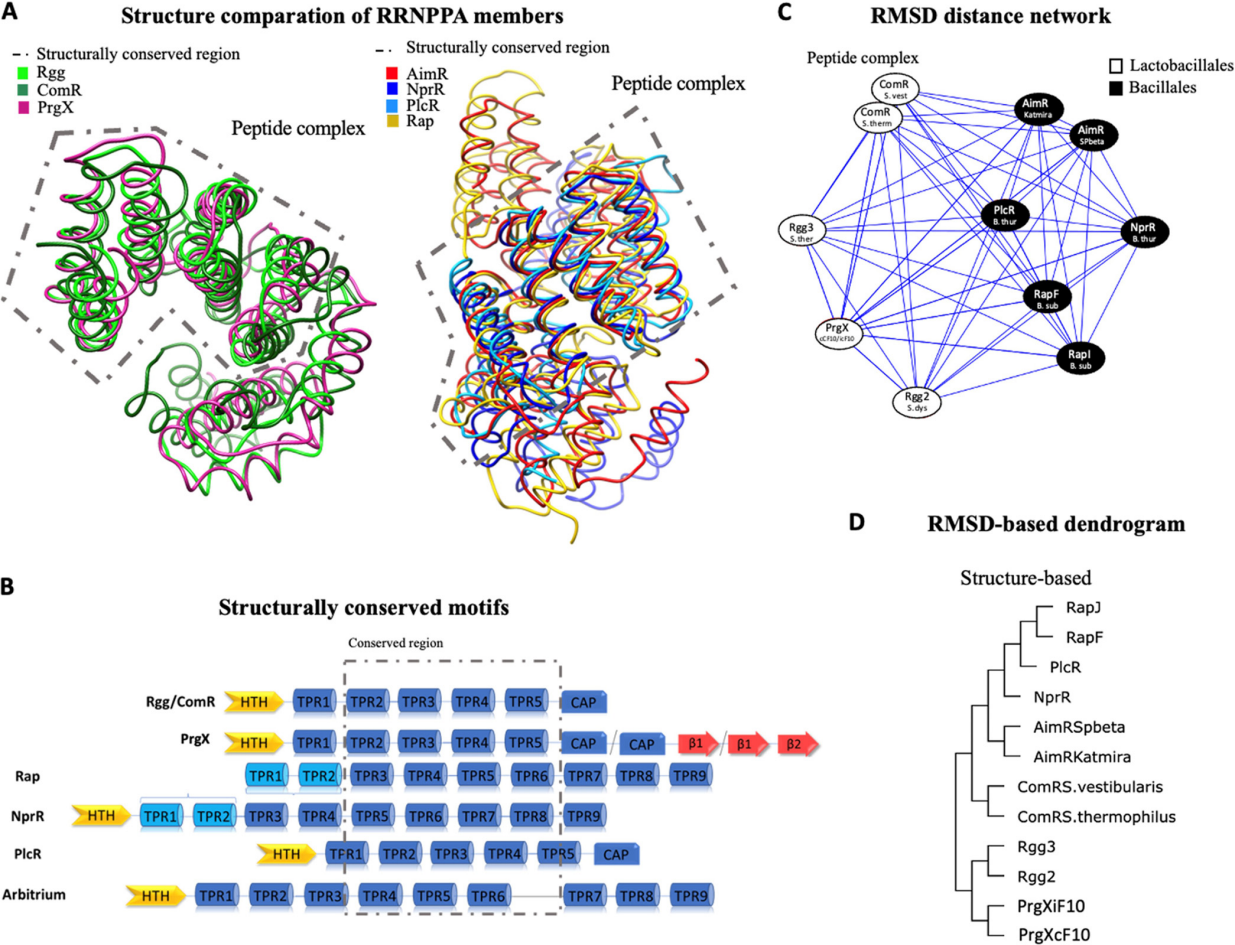
Structure-based phylogenetic reconstruction versus sequence-based phylogeny. (A) At the left, structural superposition of TPR-like domains in the peptide-bound conformation from ComR (PDB code 5JUB), Rgg (PDB code 4YV9), and PrgX (PDB code 2AXZ) is shown. At the right, structural superposition of TPR domains in the peptide-bound conformation from AimR (PDB code 6HP5), Rap (PDB code 4I9C), PlcR (PDB code 3U3W), and NprR (PDB code 4GPK) is shown. (B) Diagram representation of RRNPPA members highlighting the structurally conserved domains, which implies the following: TPR3, -4, -5, and -6 in the case of Rap; TPR5, -6, -7, and -8 in NprR; TPR2, -3, and -4, the second helix of TPR1, and first helix of TPR5 in PlcR; TPR4, -5, -6, and -7 in AimR; and TPR2, -3, -4, and -5 in the case of ComR, Rgg, and PrgX. (C) RMSD distance network from computed RMSD distances of TPR domains of representative structures of each subfamily of RRNPPA proteins in the peptide-bound state, separating those from *Bacillales* and those from *Lactobacillales*. The structures used for the analysis were ComR_S. thermophilus_ (PDB code 5JUB), ComR_S. vestibularis_ (PDB code 6HUA), Rgg2_S. dysgalactiae_ (PDB code 4YV9), Rgg3_S. thermophilus_ (PDB code 7JI0), PrgX_cCF10_ (PDB code 2AXZ), PrgX_iCF10_ (PDB code 2GRM), AimR_SPbeta_ (PDB code 6HP5), AimR_Katmira_ (PDB code 6S7L), RapF_B. subtilis_ (PDB code 4I9C), RapJ_B. subtilis_ (PDB code 4GYO), PlcR_B. thuringiensis_ (PDB code 3U3W), and NprR_B. thuringiensis_ (PDB code 4GPK). (D) Structured-based dendrogram computed from RMSD distances in structural alignment using conserved TPR domains (see PDB codes above) using the online tool mTM-align (64).

To study the evolution of the RRNPPA proteins, we took the hits of each subfamily, aligned them, and inferred their phylogeny. The existence of low sequence identity and different protein architectures across the subfamilies complicates their inclusion in a single phylogeny. To minimize this problem, we focused on the conserved domains of the proteins that provide better multiple alignments. We first used the HTH DNA binding domain, whose function is conserved in the different subfamilies. However, these domains are small, providing little phylogenetic information, and are absent from Rap proteins, excluding them from the analysis. As an alternative, we sought to analyze TPR domains. However, these are present in multiple copies, complicating the identification of the regions of homology between proteins. To precisely identify these regions, we analyzed publicly available structures of RRNPPA members in its apo conformation (without peptide) and bounded to the peptide. The analysis of the structures revealed that three of the TPR domains are conserved from the structural point of view in the apo conformation (4 TPR domains in a peptide-bound state) (Fig. 3A and 3B). Hence, we focused on these three TPR domains. We built an interaction network from the distance matrix calculated from root mean square deviation (RMSD) of atomic positions between structures. We used peptide-bound structures for this analysis, because the presence of the peptide stabilizes the protein, closing the TPR domains, and allows for better structural alignments. Interestingly, the structure-based dendrogram and the distance network revealed coherent results: in both, the proteins from the same subfamily and subfamily members for *Bacillales* and *Lactobacillales* clustered together (Fig. 3C and D). Proteins with similar architectures (such as NprR and Rap proteins, which are the only ones to have a 3HB domain) clustered together as well, even if those domains were not included in the analysis. A structure-based dendrogram (computed from RMSD distances) showed similar results (Fig. 3C and D).

**FIG 4:**
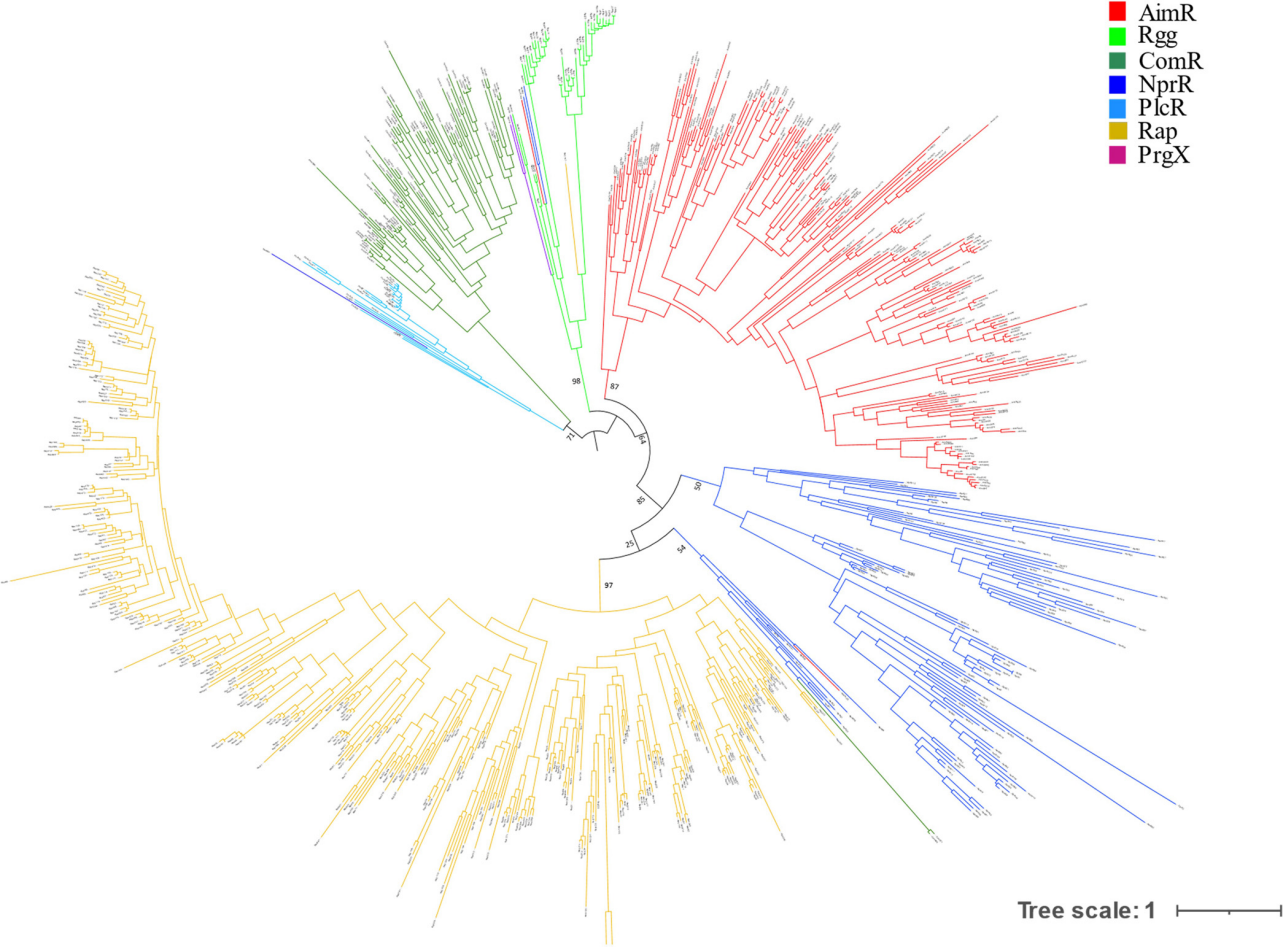
Sequence-based phylogeny of RRNPPA proteins found during an HMM search. The analysis was performed using nonredundant proteins (less than 95% identity in 95% coverage). The tree was built using IQ-TREE (see Materials and Methods). Branches are colored according to subfamily.

Since three TPR domains are structurally conserved, we took the hits of each subfamily, selected the structurally conserved TPR domains, aligned them, and inferred their phylogeny. This resulted in alignments that were longer, more conserved, and had fewer gaps than those of HTH (see Fig. S2 in the supplemental material). We used it to make a phylogenetic tree. Intriguingly, we found two small polyphyletic clades in the tree, one close to Rgg and the other close to the Rap cluster, where almost every protein present is from a different RRNPPA subfamily (Fig. 4). We cannot explain this result, but we suspect that it is a reconstruction artifact. The structure-based analyses and these phylogenetic reconstruction have key similarities: proteins from the same subfamily cluster together, and those from *Bacillales* and *Lactobacillales* make different clusters in the tree. Also, NprR and Rap proteins cluster together, in agreement with the structure-based analysis, and the phylogenetic tree suggests that Rap derived from NprR proteins.

10.1128/mbio.02514-22.2 FIG S1 Coverage against domain E value in the detection of RRNPPA homologs performing hmmsearch. Shown for the different subfamilies of the RRNPPA family (from A to G) are the coverage and domain E value obtained for the hits using hmmsearch. Shown in blue lines are the thresholds that we set to determine if the hit was an actual homolog. Download FIG S1, PDF file, 0.2 MB. Copyright © 2022 Felipe-Ruiz et al. 2022 Felipe-Ruiz et al. https://creativecommons.org/licenses/by/4.0/ This content is distributed under the terms of the Creative Commons Attribution 4.0 International license.

10.1128/mbio.02514-22.3 FIG S2 Comparison between trees constructed from HTH DNA binding domain, structurally conserved TPR domains, and complete proteins. Phylogeny from RRNPPA proteins found during the HMM search. The analysis was performed using nonredundant proteins (less than 95% identity in 95% coverage). The tree was built using alignment of specific domains or full proteins using IQ-TREE (see Materials and Methods). Branches were colored depending on the subfamily. Download FIG S2, PDF file, 0.7 MB. Copyright © 2022 Felipe-Ruiz et al. 2022 Felipe-Ruiz et al. https://creativecommons.org/licenses/by/4.0/ This content is distributed under the terms of the Creative Commons Attribution 4.0 International license.

### Pheromone identification and characterization.

The known pheromone genes are next to the RRNPPA gene (except for PrgX [41]). This simplifies their identification. Hence, we searched for genes encoding small peptides close to the RRNPPA genes and with evidence of being secreted by the standard secretory pathways. This analysis was performed for all the subfamilies, and revealed 3785 peptides for the subfamilies Rap, AimR, NprR, and PlcR (Table 2). We found no evidence of genes encoding propeptides next to genes encoding PrgX, Rgg, and ComR receptors. In the case of Rgg and ComR, the previously described propeptides of these subfamilies, called short hydrophobic peptides (SHPs), are smaller than the other propeptides, and they are secreted by PptAB, an ABC-type transporter (42). Their small size and different secretion mechanisms might explain why they were missed. Hence, we searched for them using a complementary approach. We identified all small ORFs upstream of the gene, translated them, and then looked for homologs of previously described mature peptides. In the case of PrgX, we performed the same search but using the complete plasmid carrying PrgX homologs, finding propeptides in 16 out of the18 plasmids analyzed.

**TABLE 2:** Number of peptides and percentage of RRNPPA hits where peptide candidate was found

RRNPPA subfamily	% of peptide found	No. of peptides
AimR	63.75	482
ComR	62.4	394
NprR	40	113
PlcR	89.36	126
Rap	74.74	3,064
Rgg	100	658
PrgX	88	16

**FIG 5:**
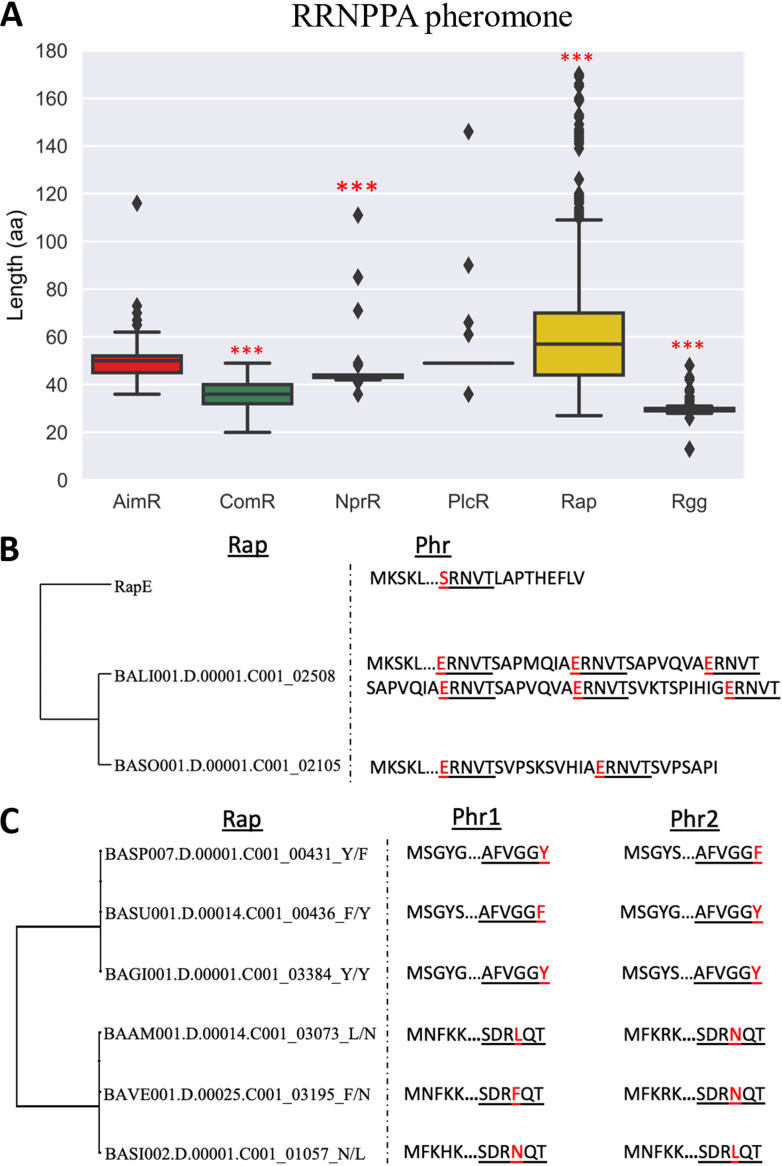
Pheromone precursor characterization in the RRNPPA subfamilies and models for pheromone duplication. (A) Box diagram for the length of the peptide precursor of the pheromone. Interquartile ranges are shown; the horizontal line within each box represents the median, and the whiskers extend out from lower to upper fence. Nonparametric Kruskal-Wallis analysis was performed to compare differences between AimR, Rap, Rgg, ComR, NprR, and PlcR peptides. ***, *P* ≤ 7.92e−7, *P* ≤ 3.63e−75, *P* ≤ 1.27e−63, and *P* ≤ 6.32e−22 for Rap, Rgg, ComR, and NprR, respectively. (B) Phylogenetic tree of six specific Rap variants with two different propeptide candidates and their respective Phr sequences. Sequence alignment was performed using MAFFT version 7.471 (58); the resulting alignment was used to create a maximum likelihood phylogenetic tree using IQ-TREE (see Materials and Methods). (C) Phylogenetic tree of three specific Rap variants with internally repeated propeptides and their respective Phr sequences. Alignment was performed and the tree was created as described above. The first amino acids of the Phr sequence are omitted. Underlined are repeated sequences of the putative Phr autoinducer. Marked in red is a putative autoinducer sequence that deviates from the others.

Previous studies revealed that one RRNPPA propeptide can contain one or two copies of mature peptides (35). To quantify this trait, we characterized the length of the propeptides. Rap proteins have longer propeptides than the other subfamilies, while ComR and Rgg have, as expected, smaller propeptides (Fig. 5A). The identification and characterization of internally pseudorepeated peptides are not trivial: it must be done one by one, looking for pseudorepetitions of the last 5 amino acids in the propeptide or repetitions of sequences similar to other described mature peptides. This analysis is not feasible for all 4,842 propeptides. Instead, we searched for internally pseudorepeated peptides in the atypically large propeptides, i.e., those classified as outliers in Fig. 5A. A total of 6.7% of these large propeptides carried more than one copy of the predicted mature peptide (either identical or pseudorepeated) (Table 3). We did not identify duplicates among the propeptides of the Rgg and ComR subfamilies. It is possible that the export pathways of these subfamilies are incompatible with peptide duplication or elongation. In contrast, multiple copies of mature peptide were common in propeptides associated with the AimR, NprR, Rap, and PlcR subfamilies.

**TABLE 3:** Proportion of pseudorepeated propeptides among atypically large ones^*a*^

RRNPPA subfamily	Pseudorepeated propeptides found	Identically repeated propeptides found	% of propeptides with repetitions (no./total)
AimR	✓	✓	27 (3/11)
ComR	✗	✗	0 (0/394)
NprR	✗	✓✓	100 (17/17)
PlcR	✓	✓	20 (3/15)
Rap	✓	✓	1.6 (5/311)
Rgg	✗	✗	0 (0/658)
PrgX	✗	✗	0 (0/16)

a ✓, presence; ✗, absence; ✓✓, high abundance.

The different copies of the mature peptides included in a single propeptide can be identical or dissimilar. The former corresponds to propeptides producing more identical peptides than the average gene. The latter corresponds to propeptides producing different peptides, i.e., different messages. The subfamilies AimR, NprR, PlcR, and Rap had up to six copies of the same mature peptide in their propeptides. Such genes were also found in MGEs such as prophages (Fig. 5B). Dissimilar peptides within the same propeptide were found in atypically large genes of the AimR, Rap, and PlcR subfamilies (Table 3). In some cases, the propeptide has multiple identical copies of the mature peptide and one copy different from the others.

Finally, several members of Rap subfamily have more than one neighboring gene encoding a propeptide. We found 71 Rap proteins with two neighbor genes encoding putative propeptides. In 13 of these, both genes neighboring the Rap encode identical mature peptides. The remaining 58 cases concern genes encoding different mature peptides. In the latter cases, the peptides only differ in one very specific amino acid position of the mature pheromone (the fifth amino acid in one group and the sixth amino acid in other) (Fig. 5C). This accumulation of variability in one single position of the mature pheromone suggests that it may provide specificity in the pheromone-receptor recognition.

### Peptide regulation: chatterers, eavesdroppers, and other systems of signaling.

The presence of receptors with multiple copies of the gene encoding the QS pheromone led us to consider the possibility that some QS regulators could lack a peptide pheromone. These systems could be regarded as “eavesdroppers” since they recognize signals produced by other systems but do not generate a QS signal that could be recognized by others (see introduction). One could expect systems lacking propeptides to be particularly frequent in MGEs, which could use them to eavesdrop on the host or on other MGEs. To test this hypothesis, we analyzed the percentage of receptor genes lacking neighboring genes encoding propeptides. We restricted this analysis to Rap and AimR systems because these are the ones frequent in the bacterial chromosome and in MGEs (Table 4). The frequency of putative such Rap systems was high in phage-plasmids (almost 50%) but low in the other MGEs. AimR systems of plasmids and phage-plasmids rarely lack recognizable propeptides. Instead, almost 90% of the AimR systems in chromosomal regions not classed as prophage were putative eavesdroppers (Table 4). Interestingly, in the case of PrgX, which is present only in plasmids, the two systems where we could not find candidate peptides are in strains that carry a second plasmid with genes for the PrgX receptor and peptide. This suggests the existence of eavesdropper behaviors also among PrgX systems. Taken together, these results suggest that systems lacking propeptides could be very frequent. Their presence in MGEs depends on the nature of the system and of the MGE.

**TABLE 4:** Percentage of Rap and AimR genes where a propeptide candidate was detected

Protein	% in:			
	Other	Prophages	Phage-plasmids	Plasmids
Rap	74	76	56	82
AimR	11	65	100	97

### Pheromone export signal.

RRNPPA systems regulate important processes in MGEs, including the lysis-lysogeny decision or conjugation (21, 39). The steps of pheromone export and maturation of QS peptides pose specific challenges for the systems encoded in MGEs because there may not be adequate enzymes to handle them in the novel host cell. An additional complication arises from the existence of propeptides with multiple peptides, which require a specific process of maturation including cuts in the N-terminal and in the C-terminal parts of the peptides.

**FIG 6:**
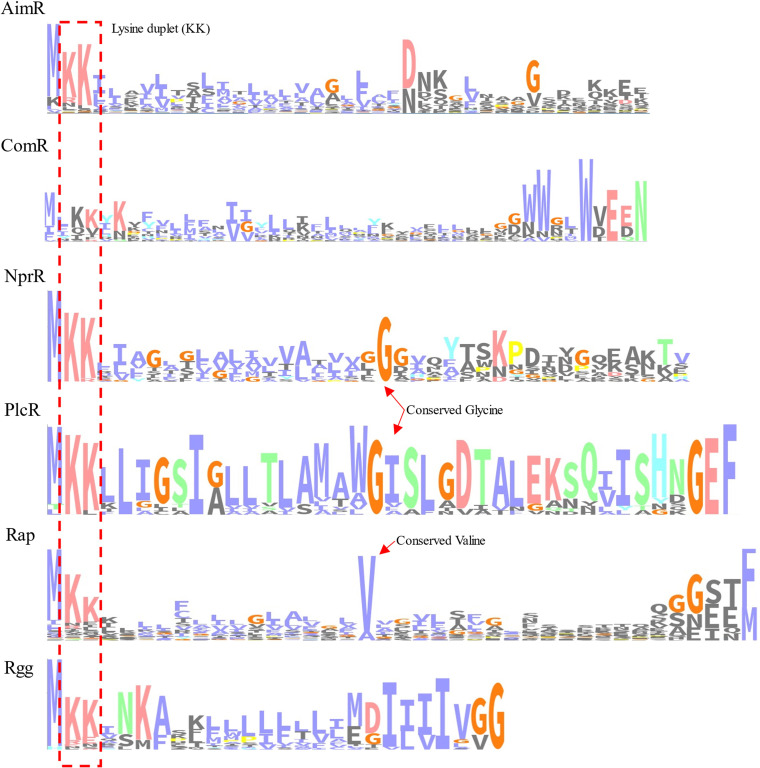
Consensus export signal for the pheromone precursor in the different RRNPPA subfamilies. Sequence logos were constructed using observed counts corresponding to the first 40 positions in the peptide alignment. A consensus color scheme depending on column composition is used. Conserved positions and lysine duplet are highlighted.

Folded and unfolded proteins are exported by Tat and Sec translocons, respectively (43). The sequence alignment of the different subfamilies of peptides revealed that the lysine duplet (KK), implicated in export via the Sec translocon, was conserved among subfamilies. Accordingly, 56% of the peptides carried a canonical KK duplet, even in those not predicted to be exported by this pathway (Rgg and ComR), suggesting that the lysine duplet is also implicated in peptide export by the PptAB transporter (Fig. 6). After the position of the lysine duplet, all the peptides showed a hydrophobic region, implicated in export (44). The shorter propeptides of ComR and Rgg (Fig. 5A) are also more hydrophobic than those of other subfamilies, even the part corresponding to the QS pheromone, which is likely to result from the different mechanisms of export and maturation used by these two systems (Fig. 6). NprR and PlcR peptides showed a conserved glycine after a hydrophobic helix, present in almost every peptide. This glycine is changed to valine in almost all Rap propeptides (Fig. 6). The implication and importance of these conserved residues are still not clear.

Once exported, the signal peptide can be processed by a signal peptidase of type I or II (SPI or SPII, respectively). Type I SPs are the predominant bacterial endopeptidases for the processing of signal peptides. They are serine proteases using a serine and a lysine to form a catalytic dyad where the serine acts as a nucleophile. Type II SPs cut signal peptides of bacterial lipoproteins presenting a well-conserved “lipobox.” This occurs by the action of two aspartate catalytic residues (45). The analysis of the 2,734 peptides (72% of the total) for which we could identify a putative mechanism of secretion using SignalP (46) showed that most of them (2,523 [92.3%]) were exported by the Sec translocon and cleaved by SPI (SEC/SPI pathway). Of the remaining 211 peptides putatively cleaved by SPII (SEC/SPII pathway), 59 (27%) were found in MGEs, which is almost twice the frequency of RRNPPA systems in such MGEs (14%, χ^2^ test; χ^2^ = 34.16; *P* = 5.06e−09). Of these 59 propeptides predicted to be secreted by SEC/SPII pathway in MGEs, 39 were encoded in plasmids, 19 in prophages, and 1 in phage-plasmids, revealing that all those MGEs also use this export system. These results suggest that SEC/SPII is important in the maturation of propeptides in the RRNPPA systems of MGEs. None of the peptides was predicted to be exported by the Tat translocon. This was expected, since this pathway is implicated in active translocation of already folded proteins, while pheromone precursors should have reduced or null secondary and tertiary structures. In the case of Rgg, ComR, and PrgX, the Eep protease could be an alternative to SPI and SPII. This protein is found in *Enterococcus* and Streptococcus and was shown to have proteinase activity on exported proteins (47).

Most of the proteins related to the SEC/SPI translocon are conserved and constitutively expressed among *Firmicutes* (48). Nevertheless, the expression of some signal peptidases is finely regulated (49), and this could complicate the control of pheromone maturation. Moreover, some systems may require novel proteases for processing their own peptides (i.e., pseudorepeated propeptides). Interestingly, some of the main components of the SEC/SPI translocon, SecA/B/Y, SipS/P, and SRP, are present in MGEs, suggesting that the latter may complement or modify the host export machinery. We found 92 homologs of these genes in prophages (69 of them SRP homologs), 164 in plasmids (81 of them SipP), and only 1 in phage-plasmids (Table S2). The relative abundance of SEC/SP proteins in MGEs was low, corresponding to less than 0.4% of all these gene families in the genomes. Among the 257 MGEs carrying homologs of the SEC/SPI translocon, 37 of them, all plasmids, also encode an RRNPPA protein (19 Rap, 17 AimR, and 1 NprR). Interestingly, all these plasmids encode homologs of SipP and 3 plasmids encode SipP, AimR, and Rap homologs. The genes encoding SipP and RRNPPA proteins were rarely colocalized in the plasmids since their distances varied from 6 to 430 genes (average of 76.6 for Rap and 28.8 genes for AimR), suggesting independent genetic regulation of RRNPPA and *sipS*.

10.1128/mbio.02514-22.5 TABLE S2 SEC/SP homologs in MGE. Download Table S2, DOCX file, 0.01 MB. Copyright © 2022 Felipe-Ruiz et al. 2022 Felipe-Ruiz et al. https://creativecommons.org/licenses/by/4.0/ This content is distributed under the terms of the Creative Commons Attribution 4.0 International license.

## DISCUSSION

In *Firmicutes*, RRNPPA is the predominant family of known QS receptors and has many important functions. Although the members of RRNPPA family share functional and structural characteristics, their low sequence similarity complicated previous studies on their origin, distribution, and diversification. While the manuscript was being finished, two preprints from the same group revealed the presence of a number of RRNPPA QS receptors in bacterial and phage genomes (50, 51). Our results are consistent with these findings. Yet, no previous study had managed to clearly separate the different families and obtain a phylogeny to guide the classification of subfamilies and study their evolution. To fill this gap, we performed structure-based and sequence-based analyses of RRNPPA systems. Our results suggest that these systems diverged early in the history of the *Bacilli* class and gave rise to systems specific to *Lactobacillales* (Rgg, ComR, and PrgX) and *Bacillales* (AimR, PlcR, NprR, and Rap). This ancestral diversification of RRNPPA systems is associated with some of the major differences in terms of receptor architecture: Rgg, ComR, and PrgX present degenerated TPR repeats. In contrast, the other proteins have canonical TPR repeats.

The systems of *Bacillales* (AimR, PlcR, NprR, and Rap) encode the QS pheromone immediately downstream of the RRNPPA gene, which is exported and processed by the SEC/SPI and SEC/SPII translocons. In contrast, alternative pheromone dispositions and maturation pathways were described for *Lactobacillales*. These differences suggest two different evolutionary scenarios for the evolution of the RRNPPA protein-pheromone pair. First, the QS propeptide could have been acquired multiple times in natural history, explaining the large differences between the peptides of *Lactobacillales* and *Bacillales*. This would imply that the common ancestor of RRNPPA systems lacked the capacity to bind QS pheromones. The transcriptional regulators MutR and GadR encoded in *Bacilli* could be representatives of such a putative ancestor system (52, 53). These two proteins lack known QS functions and show sequence similarity to Rgg proteins. Another possibility is that peptides evolved very fast from a common ancestor. This is supported by the fast evolution of the peptides of each family. Yet, this hypothesis cannot be assessed by the analysis of propeptide sequences, because these are excessively divergent.

The phylogenetic analyses informed the diversification of the domains of the proteins. The analyses at the level of structure (Fig. 3D) and sequence (Fig. 4) show that NprR and Rap proteins are closely related. This is consistent with NprR and Rap proteins having similar 3HB domains that are lacking in the other members of the RRNPPA family. Another distinctive feature between them is that Rap proteins are the only members of RRNPPA which lack an HTH DNA binding domain. The phylogenetic data suggest that this is a derived state, i.e., a trait that was acquired after these proteins diverged from their most recent common ancestor with NprR. Hence, the integration of the information on the phylogeny and domain architecture of Rap and NprR proteins suggests that the common ancestor of these proteins might resemble NprR proteins. This hypothesis is also supported by taxonomical data, since NprR systems were found in 21 different genera, while Rap proteins were found in only 3.

Our study challenges previous conclusions on the evolutionary relationships between PlcR and PrgX. Their structural similarities and the finding of putative PlcR systems in both *Lactobacillales* and *Bacillales* using DELTA-BLAST searches of the NCBI nonredundant (nr) protein database led to suggestions that PlcR was the ancestral RRNPPA protein in *Lactobacillales* and was at the origin of ComR, Rgg, and PrgX (1, 54). In this study, we found no evidence for the presence of PlcR systems in *Lactobacillales* species and the above hypothesis was not supported by the phylogenetic analyses.

The internal amplifications and the multiplication of propeptide genes may have important consequences for the function of the QS systems. Previous studies proposed that internal duplications of propeptides could facilitate the diversification of the QS signaling pheromone, by keeping one copy of the original peptide and allowing the second one to gain novel specificities (35). Here, we have revealed a diversity of processes of evolution. Some propeptides encode multiple identical copies (up to 6 copies) of the mature QS pheromone. This could increase the pheromone/receptor ratio, generating a mechanism of “chattering.” We also found propeptides including different variants of the peptides. These propeptides may facilitate the diversification of pheromones, allowing one copy to gain novel specificities and another to maintain the original function. This could have additional functional implications. For example, one peptide could evolve to regulate the cognate protein and another could interact with other systems. This mechanism could be widespread among AimR, PlcR, Rap, and NprR systems, which have many propeptides with multiple peptides. Interestingly, an alternative mechanism of producing multiple peptides was found exclusively among some elements of the Rap subfamily, which have multiple genes encoding propeptides. Depending on the similarity between these different genes, their multiplicity could result in the production of more abundant mature peptides, e.g., chattering, or in the production of diverse variants of mature peptides. These cases resemble the possible outcomes of propeptides with multiple peptides but differ from the latter in that they require the expression of multiple genes and may have simpler mechanisms of maturation (see below). The reasons for the absence of multiple propeptide ORFs in the other RRNPPA systems are not known.

The finding of propeptides with multiple copies of the active pheromone raises an additional intriguing question: how are they processed? Most QS propeptides have the active pheromone at the C terminus, needing just one proteolytic cut in its N-terminal part to generate the fully maturated pheromone (Fig. 1B). In propeptides with multiple peptides, the C-terminal one can be generated using this mechanism. However, to generate functional pheromones, all the remaining peptides from the propeptide must be maturated by sequential proteolytic cuts in the N-terminal and in the C-terminal part of the peptide. We expect that N- and C-terminal cuts require different proteases because the signal motifs and the enzymatic mechanisms are different. Hence, the presence of multiple genes for propeptides in the Rap systems could be a simple solution to produce multiple peptides without requiring alternative maturation mechanisms. How propeptides with multiple peptides are processed and maturated remains a fascinating mystery. Propeptide maturation can be a problem particularly important for MGEs since they may infect cells lacking the required maturation enzymes. Interestingly, we identified plasmids encoding simultaneously RRNPPA QS systems and homologs of the SipP family of the peptide maturation machinery. This may be a strategy to circumvent the problem, allowing the MGEs to be less dependent on the host cell machinery for peptide maturation.

Finally, our analysis confirms that RRNPPA QS systems lack peptide pheromones and suggests that these cases can be very frequent in certain subfamilies. These results should be interpreted with caution, since we cannot exclude the possibility that we failed to identify atypical propeptides. Furthermore, the data set we used is very large, but it is still far from being an exhaustive sample of the genomes of *Firmicutes*. As a case in point, two known peptides of PrgX (LTSWWGL and AIFILAS) could not be identified in our genomes even when translating all open reading frames. Still, for most families we did identify all known peptides. Hence, we suggest that the existence of systems without pheromones could be adaptive, generating systems functioning as “eavesdroppers” that recognize peptides from other systems but are not implicated in communication. Interestingly, our analysis revealed many putative eavesdroppers among the Rap and AimR systems, with an overrepresentation in the latter subfamily of systems in chromosomal regions that were not classed as prophage. The presence of AimR systems, which control phage lysis-lysogeny decision, in such regions is intriguing and suggests that such systems could be used by the bacterium or other MGEs integrated in the chromosome to response to phage decisions.

## MATERIALS AND METHODS

### Data.

We retrieved 13,513 bacterial genomes, including 11,806 plasmids, from the NCBI nonredundant (nr) RefSeq database (ftp://ftp.ncbi.nlm.nih.gov/genomes/refseq/, last accessed in May 2019). The replicons were assigned to a bacterial host species using the GenBank file.

Prophages (integrated temperate phages) of the bacterial genomes were predicted using VirSorter version 1.0.3 46 (55) with the RefSeqABVir database. The least confident predictions, i.e., categories 3 and 6, which may be prophage remnants or erroneous assignments, were excluded from the analyses.

### Construction of HMMs of RRNPPA family members.

HMM profiles were constructed for seven different subfamilies of the RRNPPA family (Rgg, ComR, Rap, NprR, PrgX, PlcR, and AimR). We collected reference validated proteins from each subfamily (1, 56, 57) (Table S1). When a subfamily had few described members (fewer than 5), we searched for homologous proteins using the BLASTP web server of BLAST+ (58). The searches were conducted on the NCBI nr protein database, with default parameters. We selected nonredundant candidates with high coverage (>80%) and sequence identity between 30% and 80%, which are usual parameters observed between members of the same subfamily in the RRNPPA family. We used as query sequences the Enterococcus faecalis plasmid pFC10 PrgX protein (GenBank accession no. WP_002366018.1), Bacillus thuringiensis serovar *israelensis* ATCC 35646 PlcR protein (GenBank accession no. 2QFC_A), Streptococcus dysgalactiae Rgg2 protein (GenBank accession no. 4YV6_A) and Streptococcus pyogenes M1 GAS ComR protein (GenBank accession no. WP_002986681.1). In the case of the selected candidates of Rgg, ComR, and PlcR subfamilies, we searched for small hydrophobic peptides downstream and upstream of the gene (see below) with potential to be the QS propeptide to ensure that they are actual members of the RRNPPA family.

10.1128/mbio.02514-22.4 TABLE S1 Thresholds set in the detection of homologs of RRNPPA proteins. Download Table S1, DOCX file, 0.01 MB. Copyright © 2022 Felipe-Ruiz et al. 2022 Felipe-Ruiz et al. https://creativecommons.org/licenses/by/4.0/ This content is distributed under the terms of the Creative Commons Attribution 4.0 International license.

Each HMM profile was built from a multiple-sequence alignment of either the full sequences or domains of the RRNPPA proteins. Sequences of each family were aligned using MAFFT version 7.471 (59) with default parameters. The alignments were used as input to the hmmbuild tool (HMMER suite version 3.3.1 [60]) for the construction of the HMM profiles.

### Detection of homologs of RRNPPA proteins.

We searched bacterial genomes for RRNPPA proteins with HMM profiles that we built for each subfamily (Rgg, ComR, Rap, NprR, PrgX, PlcR, and AimR). This was done using hmmsearch with default parameters (HMMER suite version 3.3.1 [60]). We recovered for each hit the domain E value and its coverage over the alignment. The analysis of the plots of coverage versus E value revealed the existence of a critical region where there was a huge drop in the query coverage of the hits. From this observation, we stablished a threshold to separate positive from negative hits for each subfamily (Fig. 1 and Table S1). In the case of Rgg, the model was able to find homologs of MutR and GadR (proteins closely related with Rgg without QS activity) (Fig. S1). The hits of Rgg were filtered by searching for a gene encoding a propeptide candidate near the putative *rgg* (see below). The Rgg candidates without QS propeptide were eliminated to prevent the introduction of homologs of MutR and GadR or the addition of other non-QS regulators.

We started the analysis of SEC/SPI homologs by retrieving the homologs of SecA, SecB, SecY, SRP, SipS, and SipP from UniProt (https://www.uniprot.org). These putative homologs were curated, annotated, aligned, and then used to build HMM protein profiles (as described above). These profiles were used to search for homologs in bacterial genomes (as described above).

### Identification of the putative peptide pheromone.

We searched for small (corresponding to 20 to 120 aa) open reading frames (ORFs) harboring a putative signal peptide for exportation and located immediately downstream or upstream of the RRNPPA gene. For this, we concatenated the 50 terminal bases of each RRNPPA gene with 1,000 additional bases downstream of the gene. We did the same for the first 50 bases of the RRNPPA gene with 1,000 bases upstream of the gene. These two sequences were searched for ORFs using prodigal version 2.6.3 (61), including ORFs that begin with alternative start codons (GTG or TTG). These ORFs were then searched for the presence of peptide signals for exportation using SignalP 5.0 (46) using the “Gram-positive bacteria organism group.” When we could not identify any ORFs with a valid signal using SignalP next to an RRNPPA protein, we used PrediSi (62), using the “Gram-positive bacteria organism group” and a minimal cutoff of 0.25 for the identification of a secretion signal sequence. In the case of Rgg and ComR, the previously described propeptides could not be detected by any of the methods. To test if they were present, we translated all the 6 frames in the region upstream of Rgg or ComR. All predicted peptides with more than 10 amino acids were kept for subsequent analysis. We searched each of these peptides for homology to the previously described mature peptides using BLASTP version 2.10.1 (National Center for Biotechnology Information) with default parameters; since the sequence of the mature peptides is just 8 amino acids long, only peptides with high identity (>80%) and coverage (>85%) were kept. This procedure allowed to identify many more propeptides for ComR. In the case of PrgX, the same alternative method was applied, except that since these elements are in plasmids, we analyzed the complete sequence of the plasmids carrying *prgX* genes (and not just the region contiguous to the gene). This allowed us to identify propeptides for PrgX. All RRNPPA proteins and pheromone sequences are available at Data Set S1.

10.1128/mbio.02514-22.1 DATA SET S1 Data set of sequences of RRNPPA members and pheromones analyzed. Download Data Set S1, XLSX file, 0.5 MB. Copyright © 2022 Felipe-Ruiz et al. 2022 Felipe-Ruiz et al. https://creativecommons.org/licenses/by/4.0/ This content is distributed under the terms of the Creative Commons Attribution 4.0 International license.

### Structured-based phylogeny of RRNPPA proteins.

We collected structures in the Protein Data Bank (PDB) of RRNPPA regulators in presence or in the absence of the peptide bound: ComR_S. thermophilus_ (PDB code 5JUB), ComR_S. vestibularis_ (PDB code 6HUA), Rgg2_S. dysgalactiae_ (PDB code 4YV9), Rgg3_S. thermophilus_ (PDB code 7JI0), PrgX_cCF10_ (PDB code 2AXZ), PrgX_iCF10_ (PDB code 2GRM), AimR_SPbeta_ (PDB code 6HP5), AimR_Katmira_ (PDB code 6S7L), RapF_B. subtilis_ (PDB code 4I9C), RapJ_B. subtilis_ (PDB code 4GYO), PlcR_B. thuringiensis_ (PDB code 3U3W), and NprR_B. thuringiensis_ (PDB code 4GPK) in the peptide bound state and ComR_S. thermophilus_ (PDB code 5JUF), ComR_S. suis_ (PDB code 5FD4), Rgg2_S. dysgalactiae_ (PDB code 4YV6), Rgg3_S. thermophilus_ (PDB code 6W1E), PgrX_E. faecalis_ (PDB code 2AXU), AimR_SPbeta_ (PDB code 6HP3), AimR_Katmira_ (PDB code 6S7I), RapF_B. subtilis_ (PDB code 4I9E), RapI_B. subtilis_ (PDB code 4I1A), PlcR_B. thuringiensis_ (PDB code 4FSC), and NprR_B. thuringiensis_ (PDB code 5DBK) in the absence of the QS pheromone. Molecular graphics images and structural superposition were produced using UCSF Chimera package from the Resource for Biocomputing, Visualization, and Informatics at the University of California, San Francisco (63). Structurally conserved motifs were selected for each candidate structure, and the root mean square deviation (RMSD) of atomic positions was calculated between all the structures in the presence or absence of the peptide using the online tool mTM-align (64). We constructed an interaction network from the RMSD distances using the NetworkX python package (https://networkx.org/). The structure-based dendrogram was constructed from RMSD distances using the online tool mTM-align (64).

### Phylogeny of RRNPPA homologs.

To lower the computation cost of the phylogenetic analysis, without losing too much genetic diversity, the protein sequences of RRNPPA homologs were clustered using 95% identity and 95% coverage with MMSeqs2 version 12.113e3 (65). One representative per cluster was selected to make the phylogenetic analyses. The resulting sequences (*n *= 888) were aligned using MAFFT version 7.471 (59) with default parameters. Alignments and phylogenies were performed using either the complete protein sequence, the HTH DNA binding domain, or structurally conserved TPR repetitions. To root the tree with an outgroup homolog, we built an HMM profile of the HTH DNA binding domain using the alignment of the RRNPPA sequences. We used the HMM profile to identify in bacterial genomes (as described before) proteins with similar DNA binding domains from outside the RRNPPA family. In the case of the TPR repeats, the tree was rooted at the midpoint. All the phylogenetic trees were built by maximum likelihood using IQ-TREE version 2.0.3 (66) including 1,000 ultrafast bootstraps (67) (“-bb 1000”) and using the “-s” parameter to test for the best substitution model (68) and by performing an SH-aLRT test using the “-alrt 1000” parameter (69). The trees were visualized using the iTOL program (70).

### Data availability.

The data sets generated during and/or analyzed during the current study are available in the supplemental material.

## References

[1] Neiditch MB, Capodagli GC, Prehna G, Federle MJ. 2017. Genetic and structural analyses of RRNPP intercellular peptide signaling of Gram-positive bacteria. Annu Rev Genet 51:311–333. doi:10.1146/annurev-genet-120116-023507. 28876981 PMC6588834

[2] Monnet V, Gardan R. 2015. Quorum-sensing regulators in Gram-positive bacteria: ‘cherchez le peptide.’ Mol Microbiol 97:181–184. doi:10.1111/mmi.13060. 25988215

[3] Do H, Kumaraswami M. 2016. Structural mechanisms of peptide recognition and allosteric modulation of gene regulation by the RRNPP family of quorum-sensing regulators. J Mol Biol 428:2793–2804. doi:10.1016/j.jmb.2016.05.026. 27283781 PMC4938729

[4] Gallego del Sol F, Penadés JR, Marina A. 2019. Deciphering the molecular mechanism underpinning phage arbitrium communication systems. Mol Cell 74:59–72.e3. doi:10.1016/j.molcel.2019.01.025. 30745087 PMC6458997

[5] Mashburn-Warren L, Morrison DA, Federle MJ. 2010. A novel double-tryptophan peptide pheromone controls competence in *Streptococcus* spp. via an Rgg regulator. Mol Microbiol 78:589–606. doi:10.1111/j.1365-2958.2010.07361.x. 20969646 PMC3058796

[6] Shanker E, Morrison DA, Talagas A, Nessler S, Federle MJ, Prehna G. 2016. Pheromone recognition and selectivity by ComR proteins among *Streptococcus* species. PLoS Pathog 12:e1005979. doi:10.1371/journal.ppat.1005979. 27907154 PMC5131902

[7] Voichek M, Maaß S, Kroniger T, Becher D, Sorek R. 2020. Peptide-based quorum sensing systems in *Paenibacillus polymyxa*. Life Sci Alliance 3:e202000847. doi:10.26508/lsa.202000847. 32764104 PMC7425212

[8] Kotte A-K, Severn O, Bean Z, Schwarz K, Minton NP, Winzer K. 2020. RRNPP-type quorum sensing affects solvent formation and sporulation in *Clostridium acetobutylicum*. Microbiology (Reading) 166:579–592. doi:10.1099/mic.0.000916. 32375981 PMC7376267

[9] Hoover SE, Perez AJ, Tsui H-CT, Sinha D, Smiley DL, DiMarchi RD, Winkler ME, Lazazzera BA. 2015. A new quorum-sensing system (TprA/PhrA) for *Streptococcus pneumoniae D39* that regulates a lantibiotic biosynthesis gene cluster. Mol Microbiol 97:229–243. doi:10.1111/mmi.13029. 25869931 PMC4676566

[10] Aframian N, Eldar A. 2020. A bacterial Tower of Babel: quorum-sensing signaling diversity and its evolution. Annu Rev Microbiol 74:587–606. doi:10.1146/annurev-micro-012220-063740. 32680450 PMC7611908

[11] Perchat S, Talagas A, Zouhir S, Poncet S, Bouillaut L, Nessler S, Lereclus D. 2016. NprR, a moonlighting quorum sensor shifting from a phosphatase activity to a transcriptional activator. Microb Cell 3:573–575. doi:10.15698/mic2016.11.542. 28357327 PMC5349214

[12] Grenha R, Slamti L, Nicaise M, Refes Y, Lereclus D, Nessler S. 2013. Structural basis for the activation mechanism of the PlcR virulence regulator by the quorum-sensing signal peptide PapR. Proc Natl Acad Sci USA 110:1047–1052. doi:10.1073/pnas.1213770110. 23277548 PMC3549096

[13] Dubois T, Perchat S, Verplaetse E, Gominet M, Lemy C, Aumont-Nicaise M, Grenha R, Nessler S, Lereclus D. 2013. Activity of the *Bacillus thuringiensis* NprR–NprX cell–cell communication system is co-ordinated to the physiological stage through a complex transcriptional regulation. Mol Microbiol 88:48–63. doi:10.1111/mmi.12168. 23388036

[14] Lereclus D, Agaisse H, Gominet M, Salamitou S, Sanchis V. 1996. Identification of a *Bacillus thuringiensis* gene that positively regulates transcription of the phosphatidylinositol-specific phospholipase C gene at the onset of the stationary phase. J Bacteriol 178:2749–2756. doi:10.1128/jb.178.10.2749-2756.1996. 8631661 PMC178008

[15] Agaisse H, Gominet M, Okstad OA, Kolstø AB, Lereclus D. 1999. PlcR is a pleiotropic regulator of extracellular virulence factor gene expression in *Bacillus thuringiensis*. Mol Microbiol 32:1043–1053. doi:10.1046/j.1365-2958.1999.01419.x. 10361306

[16] Gohar M, Faegri K, Perchat S, Ravnum S, Økstad OA, Gominet M, Kolstø A-B, Lereclus D. 2008. The PlcR virulence regulon of *Bacillus cereus*. PLoS One 3:e2793. doi:10.1371/journal.pone.0002793. 18665214 PMC2464732

[17] Dubois T, Faegri K, Perchat S, Lemy C, Buisson C, Nielsen-LeRoux C, Gohar M, Jacques P, Ramarao N, Kolstø A-B, Lereclus D. 2012. Necrotrophism is a quorum-sensing-regulated lifestyle in *Bacillus thuringiensis*. PLoS Pathog 8:e1002629. doi:10.1371/journal.ppat.1002629. 22511867 PMC3325205

[18] Fleuchot B, Gitton C, Guillot A, Vidic J, Nicolas P, Besset C, Fontaine L, Hols P, Leblond-Bourget N, Monnet V, Gardan R. 2011. Rgg proteins associated with internalized small hydrophobic peptides: a new quorum-sensing mechanism in streptococci. Mol Microbiol 80:1102–1119. doi:10.1111/j.1365-2958.2011.07633.x. 21435032

[19] Chang JC, LaSarre B, Jimenez JC, Aggarwal C, Federle MJ. 2011. Two group A streptococcal peptide pheromones act through opposing Rgg regulators to control biofilm development. PLoS Pathog 7:e1002190. doi:10.1371/journal.ppat.1002190. 21829369 PMC3150281

[20] Chandler JR, Dunny GM. 2004. Enterococcal peptide sex pheromones: synthesis and control of biological activity. Peptides 25:1377–1388. doi:10.1016/j.peptides.2003.10.020. 15374642

[21] Erez Z, Steinberger-Levy I, Shamir M, Doron S, Stokar-Avihail A, Peleg Y, Melamed S, Leavitt A, Savidor A, Albeck S, Amitai G, Sorek R. 2017. Communication between viruses guides lysis–lysogeny decisions. Nature 541:488–493. doi:10.1038/nature21049. 28099413 PMC5378303

[22] Brady A, Quiles-Puchalt N, Gallego Del Sol F, Zamora-Caballero S, Felipe-Ruíz A, Val-Calvo J, Meijer WJJ, Marina A, Penadés JR. 2021. The arbitrium system controls prophage induction. Curr Biol 31:5037–5045.e3. doi:10.1016/j.cub.2021.08.072. 34562384 PMC8612738

[23] Bruce JB, Lion S, Buckling A, Westra ER, Gandon S. 2021. Regulation of prophage induction and lysogenization by phage communication systems. Curr Biol 31:5046–5051.e7. doi:10.1016/j.cub.2021.08.073. 34562385 PMC8612742

[24] van Gestel J, Bareia T, Tenennbaum B, Dal Co A, Guler P, Aframian N, Puyesky S, Grinberg I, D’Souza GG, Erez Z, Ackermann M, Eldar A. 2021. Short-range quorum sensing controls horizontal gene transfer at micron scale in bacterial communities. Nat Commun 12:2324. doi:10.1038/s41467-021-22649-4. 33875666 PMC8055654

[25] Bongiorni C, Ishikawa S, Stephenson S, Ogasawara N, Perego M. 2005. Synergistic regulation of competence development in *Bacillus subtilis* by two Rap-Phr systems. J Bacteriol 187:4353–4361. doi:10.1128/JB.187.13.4353-4361.2005. 15968044 PMC1151770

[26] Smits WK, Bongiorni C, Veening J-W, Hamoen LW, Kuipers OP, Perego M. 2007. Temporal separation of distinct differentiation pathways by a dual specificity Rap-Phr system in *Bacillus subtilis*. Mol Microbiol 65:103–120. doi:10.1111/j.1365-2958.2007.05776.x. 17581123

[27] Crespo I, Bernardo N, Miguel-Arribas A, Singh PK, Luque-Ortega JR, Alfonso C, Malfois M, Meijer WJJ, Boer DR. 2020. Inactivation of the dimeric RappLS20 anti-repressor of the conjugation operon is mediated by peptide-induced tetramerization. Nucleic Acids Res 48:8113–8127. doi:10.1093/nar/gkaa540. 32658272 PMC7430634

[28] Bose B, Grossman AD. 2011. Regulation of horizontal gene transfer in *Bacillus subtilis* by activation of a conserved site-specific protease. J Bacteriol 193:22–29. doi:10.1128/JB.01143-10. 21036995 PMC3019953

[29] Brady A, Felipe-Ruiz A, Gallego del Sol F, Marina A, Quiles-Puchalt N, Penadés JR. 2021. Molecular basis of lysis–lysogeny decisions in Gram-positive phages. Annu Rev Microbiol 75:563–581. doi:10.1146/annurev-micro-033121-020757. 34343015

[30] Bernard C, Li Y, Lopez P, Bapteste E. 2021. Beyond arbitrium: identification of a second communication system in *Bacillus* phage phi3T that may regulate host defense mechanisms. ISME J 15:545–549. doi:10.1038/s41396-020-00795-9. 33028977 PMC8027211

[31] Yi L, Dong X, Grenier D, Wang K, Wang Y. 2021. Research progress of bacterial quorum sensing receptors: classification, structure, function and characteristics. Sci Total Environ 763:143031. doi:10.1016/j.scitotenv.2020.143031. 33129525

[32] Gallego del Sol F, Marina A. 2013. Structural basis of Rap phosphatase inhibition by Phr peptides. PLoS Biol 11:e1001511. doi:10.1371/journal.pbio.1001511. 23526880 PMC3601957

[33] Kozlowicz BK, Bae T, Dunny GM. 2004. *Enterococcus faecalis* pheromone-responsive protein PrgX: genetic separation of positive autoregulatory functions from those involved in negative regulation of conjugative plasmid transfer. Mol Microbiol 54:520–532. doi:10.1111/j.1365-2958.2004.04286.x. 15469521

[34] Bae T, Kozlowicz B, Dunny GM. 2002. Two targets in pCF10 DNA for PrgX binding: their role in production of Qa and prgX mRNA and in regulation of pheromone-inducible conjugation. J Mol Biol 315:995–1007. doi:10.1006/jmbi.2001.5294. 11827471

[35] Even-Tov E, Bendori SO, Pollak S, Eldar A. 2016. Transient duplication-dependent divergence and horizontal transfer underlie the evolutionary dynamics of bacterial cell–cell signaling. PLoS Biol 14:e2000330. doi:10.1371/journal.pbio.2000330. 28033323 PMC5199041

[36] Pottathil M, Lazazzera BA. 2003. The extracellular Phr peptide-Rap phosphatase signaling circuit of *Bacillus subtilis*. Front Biosci 8:32–45. doi:10.2741/913. 12456319

[37] Parashar V, Jeffrey PD, Neiditch MB. 2013. Conformational change-induced repeat domain expansion regulates Rap phosphatase quorum-sensing signal receptors. PLoS Biol 11:e1001512. doi:10.1371/journal.pbio.1001512. 23526881 PMC3601965

[38] Perego M. 1997. A peptide export-import control circuit modulating bacterial development regulates protein phosphatases of the phosphorelay. Proc Natl Acad Sci USA 94:8612–8617. doi:10.1073/pnas.94.16.8612. 9238025 PMC23044

[39] Dunny GM, Berntsson RP-A. 2016. Enterococcal sex pheromones: evolutionary pathways to complex, two-signal systems. J Bacteriol 198:1556–1562. doi:10.1128/JB.00128-16. 27021562 PMC4959283

[40] Guglielmini J, Quintais L, Garcillán-Barcia MP, de la Cruz F, Rocha EPC. 2011. The repertoire of ICE in prokaryotes underscores the unity, diversity, and ubiquity of conjugation. PLoS Genet 7:e1002222. doi:10.1371/journal.pgen.1002222. 21876676 PMC3158045

[41] Ruhfel R, Manias D, Dunny G. 1993. Cloning and characterization of a region of the *Enterococcus faecalis* conjugative plasmid, Pcf10, encoding a sex pheromone-binding function. J Bacteriol 175:5253–5259. doi:10.1128/jb.175.16.5253-5259.1993. 8349565 PMC204993

[42] Chang JC, Federle MJ. 2016. PptAB exports Rgg quorum-sensing peptides in *Streptococcus*. PLoS One 11:e0168461. doi:10.1371/journal.pone.0168461. 27992504 PMC5167397

[43] Natale P, Brüser T, Driessen AJM. 2008. Sec- and Tat-mediated protein secretion across the bacterial cytoplasmic membrane—distinct translocases and mechanisms. Biochim Biophys Acta 1778:1735–1756. doi:10.1016/j.bbamem.2007.07.015. 17935691

[44] Beckwith J. 2013. The Sec-dependent pathway. Res Microbiol 164:497–504. doi:10.1016/j.resmic.2013.03.007. 23538404 PMC3706482

[45] Tjalsma H, Bolhuis A, Jongbloed JDH, Bron S, van Dijl JM. 2000. Signal peptide-dependent protein transport in *Bacillus subtilis*: a genome-based survey of the secretome. Microbiol Mol Biol Rev 64:515–547. doi:10.1128/MMBR.64.3.515-547.2000. 10974125 PMC99003

[46] Almagro Armenteros JJ, Tsirigos KD, Sønderby CK, Petersen TN, Winther O, Brunak S, von Heijne G, Nielsen H. 2019. SignalP 5.0 improves signal peptide predictions using deep neural networks. Nat Biotechnol 37:420–423. doi:10.1038/s41587-019-0036-z. 30778233

[47] Cheng D, Lv H, Yao Y, Cheng S, Huang Q, Wang H, Liu X, Bae T, Li M, Liu Q. 2020. Roles of the site 2 protease Eep in *Staphylococcus aureus*. J Bacteriol 202:e00046-20. doi:10.1128/JB.00046-20. 32457050 PMC7348552

[48] Schneewind O, Missiakas D. 2014. Sec-secretion and sortase-mediated anchoring of proteins in Gram-positive bacteria. Biochim Biophys Acta 1843:1687–1697. doi:10.1016/j.bbamcr.2013.11.009. 24269844 PMC4031296

[49] Tjalsma H, Noback MA, Bron S, Venema G, Yamane K, van Dijl JM. 1997. *Bacillus subtilis* contains four closely related type I signal peptidases with overlapping substrate specificities. Constitutive and temporally controlled expression of different sip genes. J Biol Chem 272:25983–25992. doi:10.1074/jbc.272.41.25983. 9325333

[50] Bernard C, Li Y, Lopez P, Bapteste E. 2021. Large-scale identification of viral quorum sensing systems reveals density-dependent sporulation-hijacking mechanisms in bacteriophages. bioRxiv doi:10.1101/2021.07.15.452460.

[51] Bernard C, Li Y, Bapteste E, Lopez P. 2021. RRNPP_detector: a tool to detect RRNPP quorum sensing systems in chromosomes, plasmids and phages of gram-positive bacteria. bioRxiv doi:10.1101/2021.08.18.456871.

[52] Sanders JW, Leenhouts K, Burghoorn J, Brands JR, Venema G, Kok J. 1998. A chloride-inducible acid resistance mechanism in *Lactococcus lactis* and its regulation. Mol Microbiol 27:299–310. doi:10.1046/j.1365-2958.1998.00676.x. 9484886

[53] Kreth J, Merritt J, Bordador C, Shi W, Qi F. 2004. Transcriptional analysis of mutacin I (mutA) gene expression in planktonic and biofilm cells of *Streptococcus mutans* using fluorescent protein and glucuronidase reporters. Oral Microbiol Immunol 19:252–256. doi:10.1111/j.1399-302X.2004.00148.x. 15209996

[54] Declerck N, Bouillaut L, Chaix D, Rugani N, Slamti L, Hoh F, Lereclus D, Arold ST. 2007. Structure of PlcR: insights into virulence regulation and evolution of quorum sensing in Gram-positive bacteria. Proc Natl Acad Sci USA 104:18490–18495. doi:10.1073/pnas.0704501104. 17998541 PMC2141804

[55] Roux S, Enault F, Hurwitz BL, Sullivan MB. 2015. VirSorter: mining viral signal from microbial genomic data. PeerJ 3:e985. doi:10.7717/peerj.985. 26038737 PMC4451026

[56] Rocha-Estrada J, Aceves-Diez AE, Guarneros G, de la Torre M. 2010. The RNPP family of quorum-sensing proteins in Gram-positive bacteria. Appl Microbiol Biotechnol 87:913–923. doi:10.1007/s00253-010-2651-y. 20502894

[57] Stokar-Avihail A, Tal N, Erez Z, Lopatina A, Sorek R. 2019. Widespread utilization of peptide communication in phages infecting soil and pathogenic bacteria. Cell Host Microbe 25:746–755.e5. doi:10.1016/j.chom.2019.03.017. 31071296 PMC6986904

[58] Camacho C, Coulouris G, Avagyan V, Ma N, Papadopoulos J, Bealer K, Madden TL. 2009. BLAST+: architecture and applications. BMC Bioinformatics 10:421. doi:10.1186/1471-2105-10-421. 20003500 PMC2803857

[59] Katoh K, Standley DM. 2013. MAFFT multiple sequence alignment software version 7: improvements in performance and usability. Mol Biol Evol 30:772–780. doi:10.1093/molbev/mst010. 23329690 PMC3603318

[60] Eddy SR. 2011. Accelerated profile HMM searches. PLoS Comput Biol 7:e1002195. doi:10.1371/journal.pcbi.1002195. 22039361 PMC3197634

[61] Hyatt D, Chen G-L, LoCascio PF, Land ML, Larimer FW, Hauser LJ. 2010. Prodigal: prokaryotic gene recognition and translation initiation site identification. BMC Bioinformatics 11:119. doi:10.1186/1471-2105-11-119. 20211023 PMC2848648

[62] Hiller K, Grote A, Scheer M, Münch R, Jahn D. 2004. PrediSi: prediction of signal peptides and their cleavage positions. Nucleic Acids Res 32:W375–W379. doi:10.1093/nar/gkh378. 15215414 PMC441516

[63] Pettersen EF, Goddard TD, Huang CC, Couch GS, Greenblatt DM, Meng EC, Ferrin TE. 2004. UCSF Chimera—a visualization system for exploratory research and analysis. J Comput Chem 25:1605–1612. doi:10.1002/jcc.20084. 15264254

[64] Dong R, Pan S, Peng Z, Zhang Y, Yang J. 2018. mTM-align: a server for fast protein structure database search and multiple protein structure alignment. Nucleic Acids Res 46:W380–W386. doi:10.1093/nar/gky430. 29788129 PMC6030909

[65] Mirdita M, Steinegger M, Söding J. 2019. MMseqs2 desktop and local web server app for fast, interactive sequence searches. Bioinformatics 35:2856–2858. doi:10.1093/bioinformatics/bty1057. 30615063 PMC6691333

[66] Nguyen L-T, Schmidt HA, von Haeseler A, Minh BQ. 2015. IQ-TREE: a fast and effective stochastic algorithm for estimating maximum-likelihood phylogenies. Mol Biol Evol 32:268–274. doi:10.1093/molbev/msu300. 25371430 PMC4271533

[67] Hoang DT, Chernomor O, von Haeseler A, Minh BQ, Vinh LS. 2018. UFBoot2: improving the ultrafast bootstrap approximation. Mol Biol Evol 35:518–522. doi:10.1093/molbev/msx281. 29077904 PMC5850222

[68] Kalyaanamoorthy S, Minh BQ, Wong TKF, von Haeseler A, Jermiin LS. 2017. ModelFinder: fast model selection for accurate phylogenetic estimates. Nat Methods 14:587–589. doi:10.1038/nmeth.4285. 28481363 PMC5453245

[69] Guindon S, Dufayard J-F, Lefort V, Anisimova M, Hordijk W, Gascuel O. 2010. New algorithms and methods to estimate maximum-likelihood phylogenies: assessing the performance of PhyML 3.0. Syst Biol 59:307–321. doi:10.1093/sysbio/syq010. 20525638

[70] Letunic I, Bork P. 2016. Interactive Tree of Life (iTOL) v3: an online tool for the display and annotation of phylogenetic and other trees. Nucleic Acids Res 44:W242–W245. doi:10.1093/nar/gkw290. 27095192 PMC4987883

