# Supplementary figures and images for: Structural and Genomic Evolution of RRNPPA Systems and Their Pheromone Signaling
Source: mBio. 2022 Oct 19;13(6):e02514-22. doi: 10.1128/mbio.02514-22 (PMC9765709; doi:10.1128/mbio.02514-22)

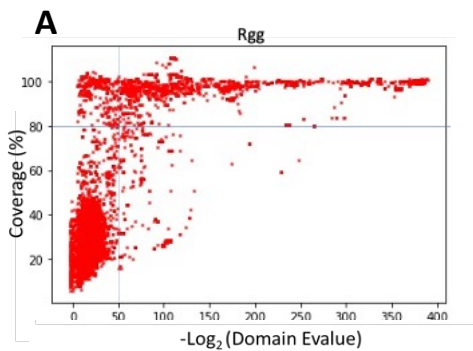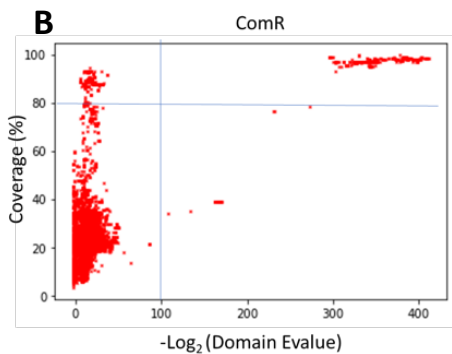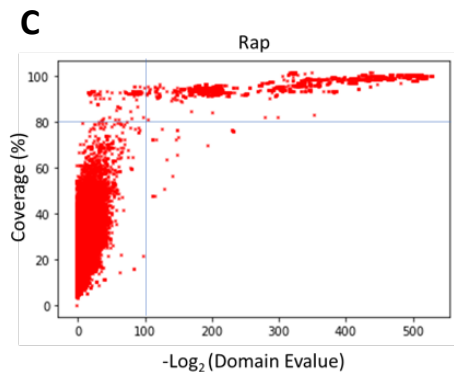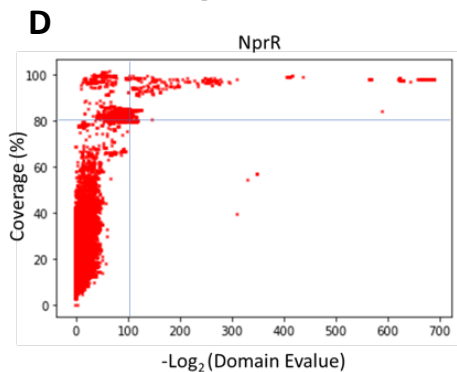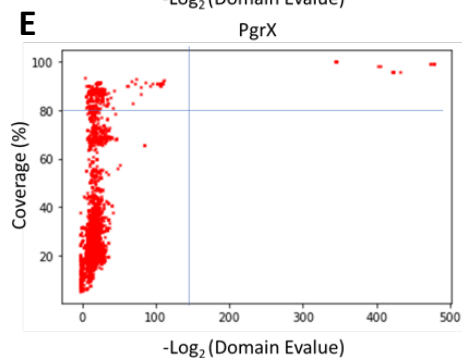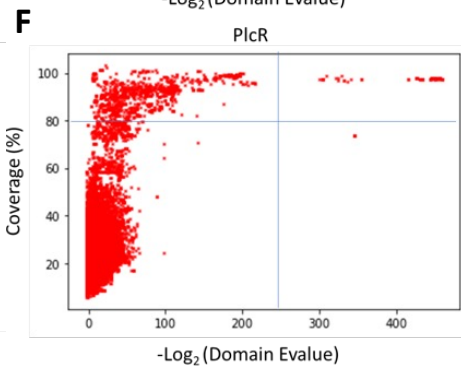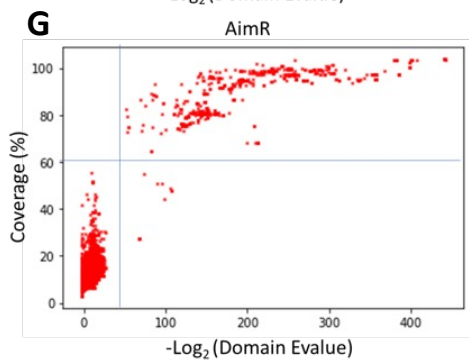

Supplement: FIG S1 [file mbio.02514-22-s0001.pdf]

HTH tree

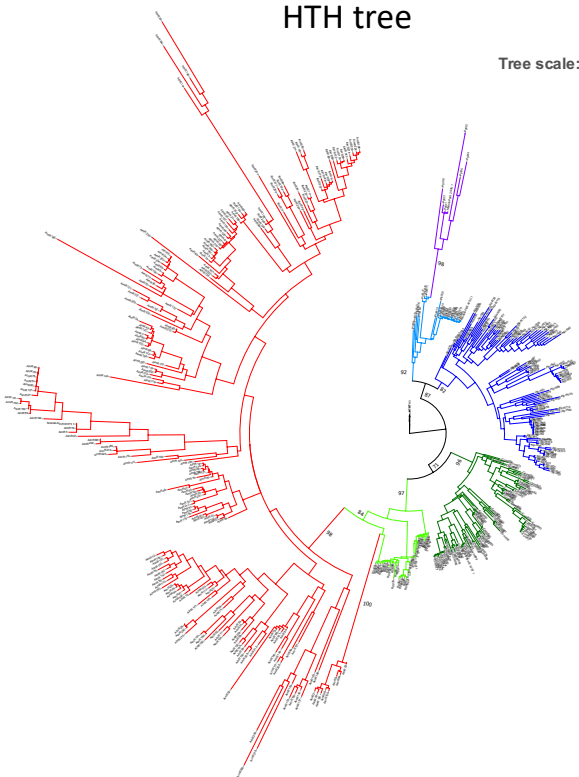

Complete proteins tree

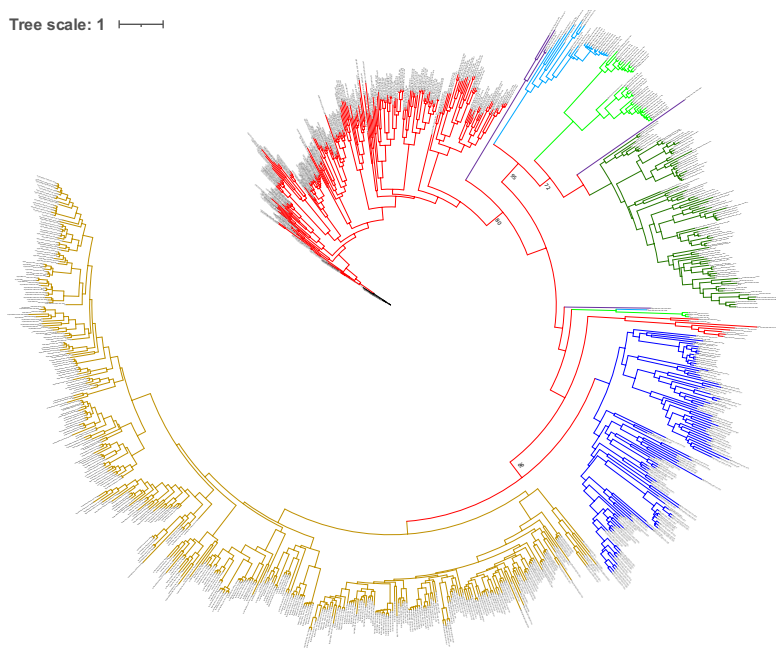

Structurally conserved TPR tree

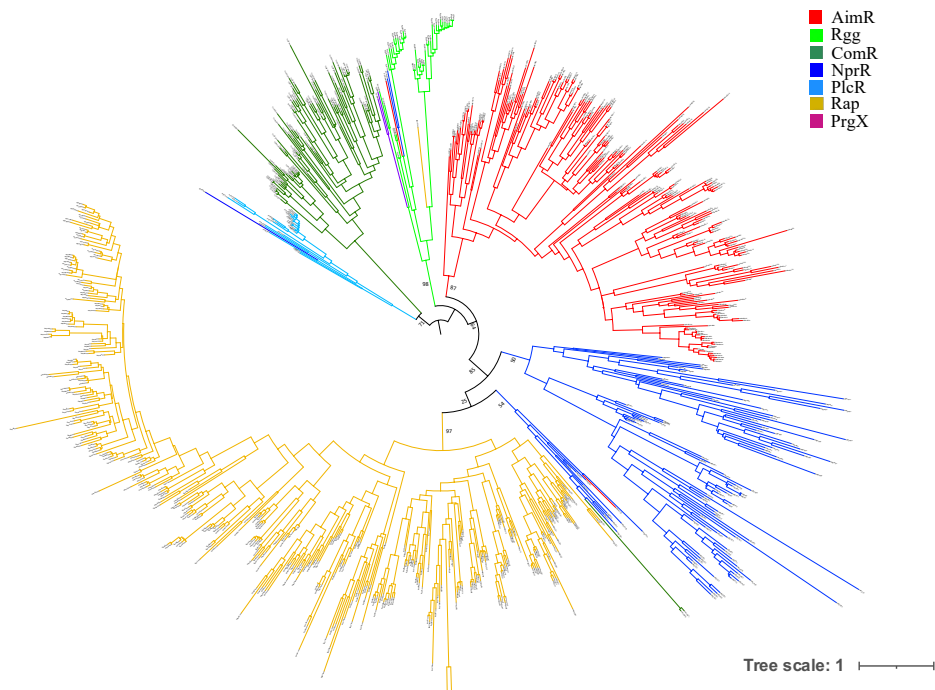

Supplement: FIG S2 [file mbio.02514-22-s0002.pdf]
