# Supplementary material for: Structural and Genomic Evolution of RRNPPA Systems and Their Pheromone Signaling
Source: mBio. 2022 Oct 19;13(6):e02514-22. doi: 10.1128/mbio.02514-22 (PMC9765709; doi:10.1128/mbio.02514-22)
Supplement: TABLE S2 [file mbio.02514-22-s0004.docx]

***Supplementary table 2: SEC/SP homologs in MGE.***

| *Protein* | *Prophage* | *Phage-Plasmids* | *Plasmids* |
| --- | --- | --- | --- |
| ***SecA*** | ***2*** | ***0*** | ***13*** |
| ***SecB*** | ***0*** | ***0*** | ***1*** |
| ***SecY*** | ***1*** | ***0*** | ***11*** |
| ***SRP*** | ***69*** | ***1*** | ***8*** |
| ***SipS*** | ***10*** | ***0*** | ***50*** |
| ***SipP*** | ***10*** | ***0*** | ***81*** |
