# Supplementary material for: Structural and Genomic Evolution of RRNPPA Systems and Their Pheromone Signaling
Source: mBio. 2022 Oct 19;13(6):e02514-22. doi: 10.1128/mbio.02514-22 (PMC9765709; doi:10.1128/mbio.02514-22)
Supplement: TABLE S1 [file mbio.02514-22-s0003.docx]

***Supplementary table 1: Thresholds set in the detection of homologs of RRNPPA proteins***

| Protein | Threshold (Domain e-value) | HMM profile | Reference sequences (GenBank accession) |
| --- | --- | --- | --- |
| AimR | 8.9e-16 | AimR.hmm | WP_053401503.1, 5ZW5_A, WP_015980882.1, WP_000878551.1, WP_139894484.1, QCW20866.1, WP_001045400.1, WP_038482237.1, WP_001996018.1, WP_000838797.1 |
| ComR | 7.9e-31 | ComR.hmm | WP_002986681.1 |
| NprR | 7.9e-31 | NprR.hmm | WP_001187961.1, P43130.1, WP_064549507.1, WP_042407712.1, WP_001187925.1, WP_073536210.1, WP_060750764.1 |
| PrgX | 7e -46 | PgrX.hmm | WP_002366018.1 |
| PlcR | 5.5e-76 | PlcR.hmm | 2QFC_A |
| Rap | 7.9e-31 | Rap.hmm | ADF59161.1, APD21157.1, NP_389125.1, NP_391550.1, NP_388259.1, NP_391519.1﻿, NP_390460.2, NP_391626.1, NP_391910.1, NP_388565.2,﻿ NP_388382.1,﻿ NP_388164.1, NP_389772.1﻿ |
| Rgg | 8.88e-16 | Rgg.hmm | 4YV6_1 |
